# Compatible solutes: the key to *Listeria's *success as a versatile gastrointestinal pathogen?

**DOI:** 10.1186/1757-4749-2-20

**Published:** 2010-12-10

**Authors:** Roy D Sleator, Colin Hill

**Affiliations:** 1 Department of Biological Sciences, Cork Institute of Technology, Rossa Avenue, Bishopstown, Cork, Ireland; 2 Alimentary Pharmabiotic Centre, University College Cork, College Road, Cork, Ireland

## Abstract

Recently we reported a role for compatible solute uptake in mediating bile tolerance and increased gastrointestinal persistence in the foodborne pathogen *Listeria monocytogenes*[1]. Herein, we review the evolution in our understanding of how these low molecular weight molecules contribute to growth and survival of the pathogen both inside and outside the body, and how this stress survival mechanism may ultimately be used to target and kill the pathogen.

## 

The Gram-positive foodborne pathogen *Listeria monocytogenes *is a causative agent of gastroenteritis [2,3] and in severe cases, listeriosis, which ranges from a mild flu-like illness to meningitis, or as infection of the foetus in pregnant women. Described as a 'Jekyll and Hyde' character [4], *L. monocytogenes *exhibits saprophytic and parasitic lifestyles; residing both in decaying plant matter in the soil [5], and as a transient inhabitant of the gastrointestinal (GI) tract of several animal species including man [6].

This physiological robustness (the ability to adapt to a variety of different environments) results from an ability to sense and respond rapidly to changes in the external environment [7]; a response mediated by a complex arsenal of genes encoding proteins linked to survival both within and outside of the host [8]. One such response, which has been the focus of significant research efforts in our laboratories, is the accumulation (either by transport [9] or synthesis [10,11]) of compatible solutes - low-molecular-weight molecules which when amassed to high intracellular concentrations help ameliorate the effects of several stressful conditions [12].

The preferred compatible solutes for the majority of bacteria and those most effective in *L. monocytogenes *are the trimethylammonium compounds; betaine, which is found in relatively high concentrations in foods of plant origin [13,14] and carnitine, which is most abundant in animal tissues [15]. Functional genomic studies, coupled with *in silico *analysis of genome sequences [8], revealed four putative compatible solute uptake systems in *L. monocytogenes*: BetL and Gbu (dedicated to betaine uptake), OpuC (which transports carnitine and to a lesser extent betaine) and OpuB which was designated as a putative compatible solute uptake system solely on the basis of sequence homology to the betaine uptake system BusA (OpuA) of *Lactococcus lactis *(Figure 1) [16].

**Figure 1:**
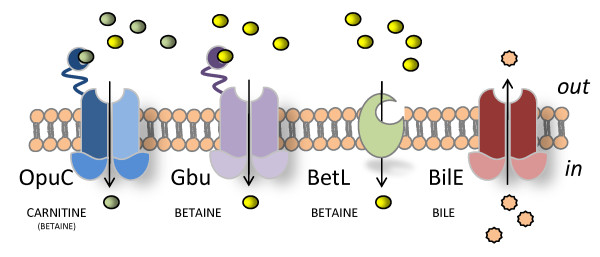
**While *in silico *analysis of the listerial genome initially identified four putative compatible solute uptake systems (BetL, Gbu, OpuC and OpuB) **[8]**, functional analysis revealed that OpuB is in fact a Bile exclusion system (hence its reincarnation as BilE)**.

Although initially identified as osmoprotective compounds (facilitating growth of the pathogen in low a_w _environments), subsequent studies revealed a multitude of beneficial effects arising from compatible solute accumulation; including protection against desiccation [17], low temperature [18] and high pressure [19] stresses encountered in foods and/or food processing environments. However, in addition to facilitating growth and survival in external environments, we have revealed a significant role for OpuC (and more specifically carnitine uptake) in enabling growth and survival of the pathogen within the host GI tract [15]. Inactivating the *opuC *gene, and thus reducing carnitine uptake, resulted in a significant reduction in the ability of *L. monocytogenes *to colonize the upper small intestine and cause subsequent systemic infection following oral inoculation. Given that the osmolarity of the gut (equivalent to 0.3 M NaCl) represents an osmotic challenge to the pathogen and that carnitine is the most abundant compatible solute in that environment (0.05 to 0.2% on a fresh weight basis), this finding was perhaps predictable. But is this the full story? Is osmotolerance alone responsible for the increased gut colonization and persistence ascribed to *opuC *in *L. monocytogenes*, or indeed *betL *when heterologously expressed in *Bifidobacterium breve*? [20]

## Double, Double, BilE and Trouble

Despite exhibiting significant sequence similarity to members of the betaine carnitine choline transporter (BCCT) family (hence the original Opu nomenclature for osmoprotectant uptake) OpuB has to date failed to display any appreciable compatible solute uptake, suggesting an alternative role for the protein. Indeed, a more detailed bioinformatic analysis of the sequence revealed two ATP-dependent bile acid permease signature sequences in the first gene of the operon [21]. Common to bile efflux pumps these motifs suggested a possible role for OpuB in listerial bile tolerance. Produced in the liver, stored interdigestively in the gall bladder and secreted into the duodenum, bile represents a far more immediate challenge to the pathogen than osmolarity and, as such, is the foremost innate immune defense mechanism of the upper small intestine [22]. Phenotypic analysis of the *in silico *findings using radiolabelled bile efflux studies revealed that OpuB did in fact function as a bile exclusion system - actively extruding bile from the bacterial cell - a phenotype which significantly modulates the virulence potential of the pathogen. That OpuB functions as a bile tolerance locus, as opposed to an osmolyte uptake system as was originally believed, led to its reincarnation as BilE (for Bile Exclusion) [21].

Notwithstanding its newly ascribed function as a bile resistance mechanism, the similarity of BilE to compatible solute uptake systems, together with the fact that it is transcriptionally regulated by the alternative sigma factor σ^B ^[22], along with BetL, Gbu and OpuC [8], suggested a common function for all four proteins; if not in osmotolerance then perhaps in bile tolerance...

In support of this hypothesis a systematic analysis of strains with mutations in the primary compatible solute uptake systems also revealed roles for OpuC, and to a lesser extent BetL, in resisting the acute toxicity of bile [1]. Furthermore, real-time gene expression profiling in the presence of bile, using a *lux *gene reporter system, revealed that both *betL *and *opuC *are induced by bile. Interestingly, while *opuC *is more highly expressed *in vitro*, *betL *exhibits higher expression levels *in vivo*. Significantly, in addition to BetL, Gbu, OpuC and BilE; σ^B ^has also been shown to regulate the expression of BSH (a bile detoxification system) and, as such, may act as the master regulator of bile tolerance in the GI tract.

## *Listeria's *Achilles heel?

The fact that compatible solutes protect *L. monocytogenes *at all stages of it lifecycle, from saprophyte to parasite, makes them a potentially important target for controlling the pathogen. Regulating the levels and/or availability of specific compatible solutes in high risk foods, e.g. baby formula (where carnitine is often added as a vitamin-like supplement), is an obvious first step [23]. While pathogen control during infection may be mediated by 'smugglin technology' - the application of toxic analogues - bactericidal compounds which, because they resemble compatible solutes, are accumulated by and ultimately kill the pathogen. Another approach which has received considerable attention in recent times is based on the patho-biotechnology concept [24-29] - the application of pathogen derived virulence or stress survival factors for the construction of improved pharmabiotic strains as biological control agents[30]. These alternative approaches to pathogen control, borne out of a clear understanding of how the pathogen adapts to its specific environment (both inside and outside the host) may ultimately provide us with a viable alternative to antibiotics for controlling old adversaries such as *L. monocytogenes*, as well as new and emerging pathogens - the so called "super bugs" [26,28].

## Competing interests

The authors declare that they have no competing interests.

## Authors' contributions

RDS and CH conceived of the study and drafted the manuscript. All authors read and approved the final manuscript.

## References

[B1] Watson D Sleator RD Casey PG Hill C Gahan CG Specific osmolyte transporters mediate bile tolerance in Listeria monocytogenes Infect Immun 2009 77 4895 4904 10.1128/IAI.00153-09 19737907 PMC2772544

[B2] Sleator RD Watson D Hill C Gahan CG The interaction between Listeria monocytogenes and the host gastrointestinal tract Microbiology 2009 155 2463 2475 10.1099/mic.0.030205-0 19542009

[B3] Sleator RD Hill C A novel role for the LisRK two-component regulatory system in listerial osmotolerance Clin Microbiol Infect 2005 11 599 601 10.1111/j.1469-0691.2005.01176.x 16008610

[B4] Gray MJ Freitag NE Boor KJ How the bacterial pathogen Listeria monocytogenes mediates the switch from environmental Dr. Jekyll to pathogenic Mr. Hyde Infect Immun 2006 74 2505 2512 10.1128/IAI.74.5.2505-2512.2006 16622185 PMC1459693

[B5] Freitag NE Port GC Miner MD Listeria monocytogenes - from saprophyte to intracellular pathogen Nat Rev Microbiol 2009 7 623 628 10.1038/nrmicro2171 19648949 PMC2813567

[B6] Sleator RD Francis GA O'Beirne D Gahan CG Hill C Betaine and carnitine uptake systems in Listeria monocytogenes affect growth and survival in foods and during infection J Appl Microbiol 2003 95 839 846 10.1046/j.1365-2672.2003.02056.x 12969299

[B7] Sleator RD Wood JM Hill C Transcriptional regulation and posttranslational activity of the betaine transporter BetL in Listeria monocytogenes are controlled by environmental salinity J Bacteriol 2003 185 7140 7144 10.1128/JB.185.24.7140-7144.2003 14645273 PMC296249

[B8] Sleator RD Gahan CG Hill C A postgenomic appraisal of osmotolerance in Listeria monocytogenes Appl Environ Microbiol 2003 69 1 9 10.1128/AEM.69.1.1-9.2003 12513970 PMC152475

[B9] Wemekamp-Kamphuis HH Wouters JA Sleator RD Gahan CG Hill C Abee T Multiple deletions of the osmolyte transporters BetL, Gbu, and OpuC of Listeria monocytogenes affect virulence and growth at high osmolarity Appl Environ Microbiol 2002 68 4710 4716 10.1128/AEM.68.10.4710-4716.2002 12324311 PMC126390

[B10] Sleator RD Gahan CG Hill C Identification and disruption of the proBA locus in Listeria monocytogenes: role of proline biosynthesis in salt tolerance and murine infection Appl Environ Microbiol 2001 67 2571 2577 10.1128/AEM.67.6.2571-2577.2001 11375165 PMC92909

[B11] Sleator RD Gahan CG Hill C Mutations in the listerial proB gene leading to proline overproduction: effects on salt tolerance and murine infection Appl Environ Microbiol 2001 67 4560 4565 10.1128/AEM.67.10.4560-4565.2001 11571156 PMC93203

[B12] Sleator RD Hill C Bacterial osmoadaptation: the role of osmolytes in bacterial stress and virulence FEMS Microbiol Rev 2002 26 49 71 10.1111/j.1574-6976.2002.tb00598.x 12007642

[B13] Sleator RD Gahan CGM O'Driscoll B Hill C Analysis of the role of betL in contributing to the growth and survival of Listeria monocytogenes LO28 Int J Food Microbiol 2000 60 261 268 10.1016/S0168-1605(00)00316-0 11016615

[B14] Sleator RD Gahan CG Abee T Hill C Identification and disruption of BetL, a secondary glycine betaine transport system linked to the salt tolerance of Listeria monocytogenes LO28 Appl Environ Microbiol 1999 65 2078 2083 10224004 10.1128/aem.65.5.2078-2083.1999 PMC91301

[B15] Sleator RD Wouters J Gahan CG Abee T Hill C Analysis of the role of OpuC, an osmolyte transport system, in salt tolerance and virulence potential of Listeria monocytogenes Appl Environ Microbiol 2001 67 2692 2698 10.1128/AEM.67.6.2692-2698.2001 11375182 PMC92926

[B16] Obis D Guillot A Gripon JC Renault P Bolotin A Mistou MY Genetic and biochemical characterization of a high-affinity betaine uptake system (BusA) in Lactococcus lactis reveals a new functional organization within bacterial ABC transporters J Bacteriol 1999 181 6238 6246 10515910 10.1128/jb.181.20.6238-6246.1999 PMC103755

[B17] Dreux N Albagnac C Sleator RD Hill C Carlin F Morris CE Nguyen-the C Glycine betaine improves Listeria monocytogenes tolerance to desiccation on parsley leaves independent of the osmolyte transporters BetL, Gbu and OpuC J Appl Microbiol 2008 104 1221 1227 10.1111/j.1365-2672.2007.03623.x 17976173

[B18] Wemekamp-Kamphuis HH Sleator RD Wouters JA Hill C Abee T Molecular and physiological analysis of the role of osmolyte transporters BetL, Gbu, and OpuC in growth of Listeria monocytogenes at low temperatures Appl Environ Microbiol 2004 70 2912 2918 10.1128/AEM.70.5.2912-2918.2004 15128551 PMC404380

[B19] Smiddy M Sleator RD Patterson MF Hill C Kelly AL Role for compatible solutes glycine betaine and L-carnitine in listerial barotolerance Appl Environ Microbiol 2004 70 7555 7557 10.1128/AEM.70.12.7555-7557.2004 15574960 PMC535178

[B20] Sheehan VM Sleator RD Hill C Fitzgerald GF Improving gastric transit, gastrointestinal persistence and therapeutic efficacy of the probiotic strain Bifidobacterium breve UCC2003 Microbiology 2007 153 3563 3571 10.1099/mic.0.2007/006510-0 17906153

[B21] Sleator RD Wemekamp-Kamphuis HH Gahan CG Abee T Hill C A PrfA-regulated bile exclusion system (BilE) is a novel virulence factor in Listeria monocytogenes Mol Microbiol 2005 55 1183 1195 10.1111/j.1365-2958.2004.04454.x 15686563

[B22] Begley M Sleator RD Gahan CG Hill C Contribution of three bile-associated loci, bsh, pva, and btlB, to gastrointestinal persistence and bile tolerance of Listeria monocytogenes Infect Immun 2005 73 894 904 10.1128/IAI.73.2.894-904.2005 15664931 PMC546953

[B23] Sleator RD Banville N Hill C Carnitine enhances the growth of Listeria monocytogenes in infant formula at 7 degrees C J Food Prot 2009 72 1293 1295 19610343 10.4315/0362-028x-72.6.1293

[B24] Sleator RD Hill C Patho-biotechnology: using bad bugs to do good things Curr Opin Biotechnol 2006 17 211 216 16459072 10.1016/j.copbio.2006.01.006

[B25] Sleator RD Hill C Patho-biotechnology; using bad bugs to make good bugs better Sci Prog 2007 90 1 14 10.3184/003685007780440530 17455762 PMC10361160

[B26] Sleator RD Hill C Battle of the bugs Science 2008 321 1294 1295 10.1126/science.321.5894.1294b 18772416

[B27] Sleator RD Hill C Engineered pharmabiotics with improved therapeutic potential Hum Vaccin 2008 4 271 274 18682694 10.4161/hv.4.4.6315

[B28] Sleator RD Hill C Designer probiotics: a potential therapeutic for Clostridium difficile? J Med Microbiol 2008 57 793 794 10.1099/jmm.0.47697-0 18480340

[B29] Sleator RD Hill C 'Bioengineered Bugs' - a patho-biotechnology approach to probiotic research and applications Med Hypotheses 2008 70 167 169 10.1016/j.mehy.2007.03.008 17452084

[B30] Culligan EP Hill C Sleator RD Probiotics and gastrointestinal disease: successes, problems and future prospects Gut Pathog 2009 1 19 10.1186/1757-4749-1-19 19930635 PMC2789095

